# Gastrointestinal bleeding after aortic surgery: a case report

**DOI:** 10.1186/1757-1626-2-9074

**Published:** 2009-11-23

**Authors:** Kohei Shitara, Ryoichi Wada

**Affiliations:** 1 Department of Clinical Oncology, Aichi Cancer Center Hospital, Nagoya, Japan; 2 Department of Gastroenterology, Kameda Medical Center, Kamogawa, Japan

## Abstract

**Introduction:**

An aortoenteric fistula is a communication between the aorta and an adjacent loop of the bowel. Here we report a case with this rare complication with typical herald bleeding.

**Case report:**

A 66-year-old man underwent elective repair of a large supra-renal abdominal aortic aneurysm and returned 6 months later to our clinic after experiencing a melena with hematochezia. The source of bleeding could not be identified by gastroscopy but the following day he vomited a large volume of blood, rapidly became haemodynamically unstable and died of hypotensive shock. A CT scan on the same day showed an increasing area of low-density soft tissue around the graft wall compared with the previous CT scan images obtained initially after the aortic repair. An aortoenteric fistula was confirmed by autopsy.

**Conclusion:**

In patients that underwent abdominal aortic surgery, both the occurrence of herald bleeding and CT findings of increasing para graft soft tissue might play a crucial role in early detection of aortoenteric fistula.

## Introduction

An aortoenteric fistula (AEF) is a communication between the aorta and an adjacent loop of the bowel. Primary fistulae occur without prior history of aortic intervention or repair and typically result from the erosion of an infected aorta into the posterior wall of the duodenum. Secondary fistulae develop after aortic intervention and typically involve a proximal suture line and/or prosthetic graft material[1]. It is a rare complication following aortic graft surgery, with a reported incidence between 0.36-4% of patients who have undergone open aortic surgery[2,3]. Here we report a case with this rare complication with typical herald bleeding.

## Case Presentation

**Figure 1:**
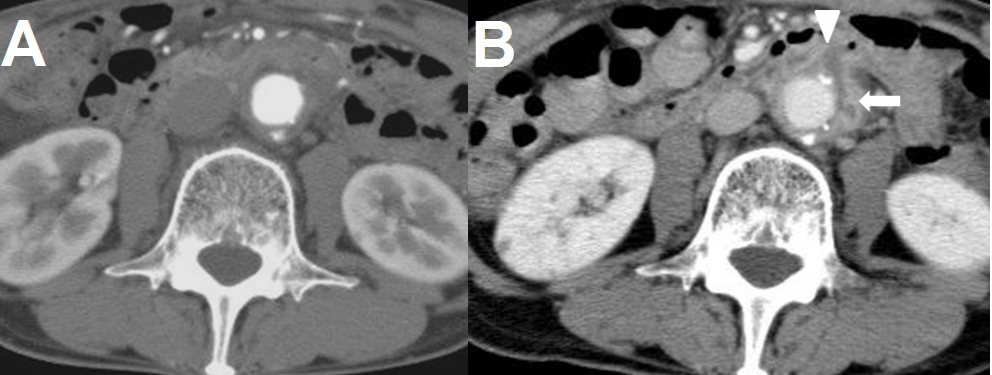
**A CT scan at 2 months after the aortic surgery**. No abnormalities were seen around the aorta. B CT scan at the fourth day after patient admission. Low-density area(*arrowhead*) had increased between the graft wall and the aneurismal wall(*arrow*), and seemed to continue to the duodenum.

**Figure 2:**
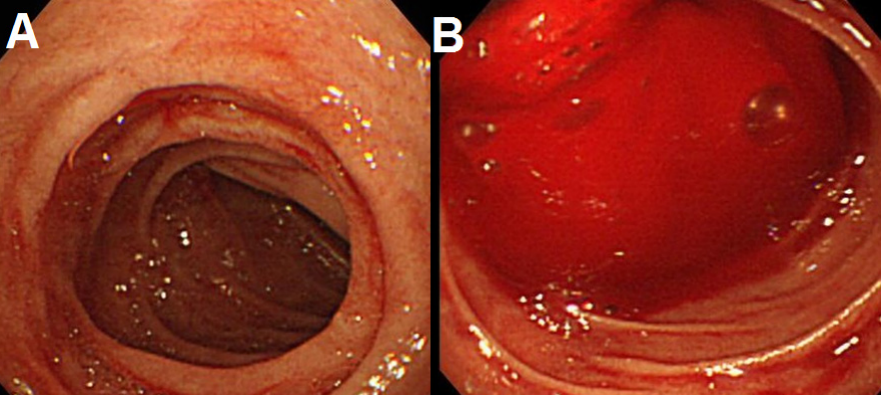
**A Gastroscopy on patient admission**. Small fresh blood stained fluids was seen in the stomach and the duodenum. C Gastroscopy at the fourth day after patient admission. Exsanguinating bleeding was seen at the third portion of the duodenum.

**Figure 3:**
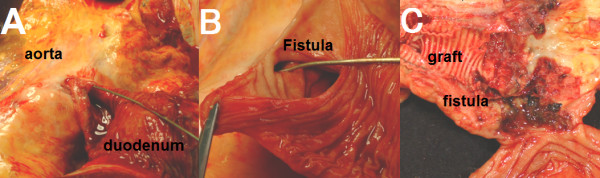
**Autopsy findings**. A Adhesion was seen between aortic wall and third portion of the duodenum. B Aorto-enteric fistula involving the third parts of the duodenum was seen in the soft tissue. C The fistula was just above the sutured site of aortic replacement. The graft wall was preserved but dirty soft tissue and thrombus was seen between the graft wall and aneurismal wall. No peritoneal or retroperitoneal hemorrhage was seen.

A 66-year-old male was admitted in *Kameda Medical Center *following a small amount of hematemesis and melena. He also had noticed another small amount of melena four and two days before admission. He had a past medical history of a supra-renal abdominal aortic aneurysm that was treated by an InterGard Y-Graft 6 months before. His postoperative course was uneventful. Follow-up computed tomography (CT) scan at 2 months showed no abnormality around aorta (Fig 1A). He had been afebrile and not noticed any symptoms until this admission. In the emergency room, his physical examination was notable for tarry stool on a rectal examination without orthostatic hypotension. No other significant abnormal findings were seen. His laboratories examinations showed white blood cell counts of 6100/μl; hemoglobin of 11.9 g/dL; platelets of 159,000/μl; blood urea nitrogen of 19 mg/dl; creatinine of 0.70 mg/dl. An emergent gastroduodenoscopy revealed small fresh blood stained fluids in the stomach and the duodenum (Fig 2A) but the site of bleeding could not be identified. Bleeding seemed to be subsided during gastroduodenoscopy. A CT scan on the following day showed low-density soft tissue area (Fig 1B*arrowhead*) between the graft and aneurismal wrap (Fig 1B*arrow*) that was not seen soon after the surgery. On the same day, he vomited large volume of blood. Emergent gastroendoscopy showed exsanguinating bleeding at the third portion of duodenum (Fig 2B). Although we consider angiography or emergent surgery for this patient but rapidly he got been haemodynamically unstable and died of hypotensive shock. Autopsy findings showed AEF involving the third parts of the duodenum (Fig 3A, B). The fistula was just above sutured site of aortic replacement and adhesion was detected around it. Although aortic wall was preserved, necrotic tissue and inflammatory change was seen around the wall (Fig 3C).

## Discussion

An AEF is a communication between the aorta and an adjacent loop of bowel[1]. The interval between aortic reconstructive surgery and the onset of gastrointestinal hemorrhage is varied. Most patients present with an initial episode of bleeding (herald bleed) followed by catastrophic hemorrhage as occurred in our case after a variable period of time. The difficulty in diagnosis of AEF was discussed in several literatures [1,3,4], and this is highlighted in our case. Although gastroduodenoscopy is important to exclude other source of bleeding, it has a low sensitivity to make the diagnosis of AEF. Abdominal CT scanning is reported to detect several abnormal findings in patients with AEF, such as increase of perigraft soft tissue, pseudoaneurysm formation, disruption of aneurismal wrap, and increased soft tissue between the graft and aneurismal wrap[3], although it has a low specificity for determining the presence of a fistula. So high suspicious should be maintained to consider the diagnosis of AEF. Curative treatment is only surgery because mortality reported 100% without surgery, although perioperative mortality also approximately 50%. For the diagnosis of the gastrointestinal bleeding in the patients following abdominal aortic surgery, especially in patients with intermittent bleeding and abnormal CT findings, AEF should be considered and early surgery should be prepared.

## Conflict of interests

The authors declare that they have no competing interests.

## Authors' contributions

KS was primarily responsible for the care for the patient and discussed the case with RW. They worked closely together in writing this report. All authors read and approved the final manuscript.

## Consent

Written informed consent was obtained from the patient' wife for publication of this case report and accompanying images. A copy of the written consent is available for review by the Editor-in-Chief of this journal.
